# Heat Tracing in a Fractured Aquifer with Injection of Hot and Cold Water

**DOI:** 10.1111/gwat.13138

**Published:** 2021-10-16

**Authors:** Richard Hoffmann, Jean‐Christophe Maréchal, Adrien Selles, Alain Dassargues, Pascal Goderniaux

**Affiliations:** ^1^ Geology and Applied Geology, Polytech Mons University of Mons Mons Belgium; ^2^ Hydrogeology and Environmental Geology, Urban And Environmental Engineering Liège University Belgium; ^3^ BRGM University of Montpellier Montpellier France; ^4^ G‐eau, UMR 183, INRAE, CIRAD, IRD, AgroParisTech, Supagro, BRGM Montpellier France; ^5^ G‐eau, UMR 183, INRAE, CIRAD, IRD, AgroParisTech, Supagro, BRGM, Indo‐French Center for Groundwater Research Hyderabad India

## Abstract

Heat as a tracer in fractured porous aquifers is more sensitive to fracture‐matrix processes than a solute tracer. Temperature evolution as a function of time can be used to differentiate fracture and matrix characteristics. Experimental hot (50 °C) and cold (10 °C) water injections were performed in a weathered and fractured granite aquifer where the natural background temperature is 30 °C. The tailing of the hot and cold breakthrough curves, observed under different hydraulic conditions, was characterized in a log–log plot of time vs. normalized temperature difference, also converted to a residence time distribution (normalized). Dimensionless tail slopes close to 1.5 were observed for hot and cold breakthrough curves, compared to solute tracer tests showing slopes between 2 and 3. This stronger thermal diffusive behavior is explained by heat conduction. Using a process‐based numerical model, the impact of heat conduction toward and from the porous rock matrix on groundwater heat transport was explored. Fracture aperture was adjusted depending on the actual hydraulic conditions. Water density and viscosity were considered temperature dependent. The model simulated the increase or reduction of the energy level in the fracture‐matrix system and satisfactorily reproduced breakthrough curves tail slopes. This study shows the feasibility and utility of cold water tracer tests in hot fractured aquifers to boost and characterize the thermal matrix diffusion from the matrix toward the flowing groundwater in the fractures. This can be used as complementary information to solute tracer tests that are largely influenced by strong advection in the fractures.

## Introduction

Groundwater flow and transport in fractured rocks occurs mainly along preferential flow paths in fractures. These fractures form a heterogeneous network, which can be defined as a domain of high permeability with fast transfer times, and where the fracture geometry controls advection and dispersion processes (Tsang et al. 1988; Tsang and Neretnieks 1998; Singhal and Gupta 2010). The fractures are usually embedded in a rock matrix, which can be considered impervious or as a porous medium with a very low permeability, where diffusive processes can dominate (Bear and Verruijt 1987; Maloszewski and Zuber 1993; Carrera et al. 1998; Kang et al. 2015). Robust decisions for sustainable management of fractured aquifers require both a realistic assessment of preferential flow paths in the fractures (Berkowitz et al. 1988; Tsang et al. 1988; Bear et al. 1993; Dassargues 2018), as well as a quantification of matrix diffusion and interactions between the rock matrix and the fractures (Kang et al. 2015; Hyman et al. 2019; Hoffmann et al. 2020; Hoffmann et al. 2021). For example, several authors interpret asymmetric tailing of observed tracer breakthrough curves in fractured rocks with matrix diffusion (Neretnieks et al. 1982; Maloszewski and Zuber 1993; Meigs and Beauheim 2001; Bodin et al. 2003; Reimus et al. 2003; Hoffmann et al. 2021). These interpretations show first that assuming the rock matrix as impervious is not very realistic at large time scales. Second, in the context of groundwater quality and geothermal applications, it is important to quantify, respectively, solute advection along with diffusion including matrix diffusion, as well as thermal convection and conduction (Neretnieks 1983; Berkowitz et al. 1988; Bear et al. 1993; Feehley et al. 2000; Barker 2010; Zhao et al. 2010).

Considering this dual domain concept for fractured rocks, weakly diffusive (*D* < 10^−9^ m^2^ s^−1^) and nonreactive tracers, such as salt or a dye, are suitable for identifying the fastest point‐to‐point solute transport pathways but are less suitable for capturing matrix diffusion processes (Domenico and Schwartz 1998; Tsang and Neretnieks 1998; Bodin et al. 2003; Hoffmann et al. 2020). Consequently, solutions based only on the advection–dispersion equation can have limited prediction capabilities for transport in fractured rocks (e.g., Bodin et al. 2003). Recent studies using stronger diffusing tracers such as dissolved gases (Hoffmann et al. 2020) or heat (Read et al. 2013; Klepikova et al. 2016a; de la Bernardie et al. 2018; de la Bernardie et al. 2019; Hoffmann et al. 2021) have shown a potential to better constrain fractured aquifer conceptualizations with matrix diffusion information. Although reliable temperature tracer experiments have been performed in porous alluvial aquifers (Wagner et al. 2014; Wildemeersch et al. 2014; Klepikova et al. 2016b; Sarris et al. 2018) and fractured porous media (Read et al. 2013; Klepikova et al. 2016a; de la Bernardie et al. 2018; de la Bernardie et al. 2019; Hoffmann et al. 2021), temperature information is still rarely analyzed by hydrogeologists, because dilution is high (Kurylyk and Irvine 2019) and the signal may be difficult to detect. Nevertheless, to understand and quantify the local matrix diffusion or mobile–immobile water interactions, the use of temperature information collected with high‐resolution sensors is very promising (Anderson 2005; Pehme et al. 2014; Irvine et al. 2015; Kurylyk and Irvine 2019). Temperature tracing can also enhance the characterization of the local fracture geometry (de la Bernardie et al. 2018) and help to interpret retardation on transport transfer times due to matrix diffusion (Hoffmann et al. 2020; Hoffmann et al. 2021).

Tracer tests using temperature are typically performed in aquifers in temperate climate zones having a natural background groundwater temperature of about 10 °C to 13 °C. The injected fluid (hot water) is thus warmer compared to the background temperature, and breakthrough curves show positive temperature anomalies. In those experiments, only heat conduction is considered in the rock matrix (immobile domain and solid matrix). Heat transfer is first observed from the fracture (mobile groundwater domain) to the matrix (immobile groundwater domain and solid matrix), and then inversely when temperature decreases in the fracture (e.g., Ma et al. 2018). These experiments are useful to quantify heat storage considering the thermal conductivity and specific heat capacity values of groundwater and rock matrices (Molson et al. 1992; Palmer et al. 1992; Bridger and Allen 2010; Luo et al. 2017; Dassargues 2018; de Schepper et al. 2019). They allow characterization of the thermal retardation within the medium and to highlight the difference of behavior compared to solute tracers (Geiger and Emmanuel 2010; Ma and Zheng 2010; Rau et al. 2012; Irvine et al. 2015, 2017; Dassargues 2018; de la Bernardie et al. 2018; Luo et al. 2018; Sarris et al. 2018; Hoffmann et al. 2019).

In the present study, injection of cold water in a fractured and weathered granite aquifer characterized by warmer background groundwater temperatures (around 30 °C) was performed. A natural background aquifer temperature of around 30 °C is typical for one‐third of the aquifers worldwide and especially in tropical regions (Benz et al. 2017). In these aquifers, thermal tracer tests have probably been underutilized for applications in aquifer characterization. In the present study, results from a hot and cold water injection performed in the same aquifer are compared. The injection of cold water produced breakthrough curves with negative thermal anomalies. The same processes are involved but a formal comparison between hot and cold tracer experiments will allow an interesting and novel comparison between the “fracture to matrix” and “matrix to fracture” heat transfer. Taking the temperature‐dependent groundwater viscosity and density into account, the experimental breakthrough curves are simulated using an identical process‐based model. This model allows to analyze the observed differences between the hot and cold water experiments and provides reliable thermal transport properties in fractured rocks. Particularly, this could be useful to investigate the impact of conduction toward or from the porous rock matrix on groundwater heat transport. Here, this is applied for a weathered/fractured granite aquifer at the meter scale, bringing new insight regarding hydraulic and thermal characterization. For this purpose, the performed temperature tracer experiments are characterized by their energy recovery rate (e.g., de la Bernardie et al. 2019) and interpreted using numerical modeling that considers multiple discrete fractures and density‐viscosity dependent flow and transport (Graf and Therrien 2005, 2007; Graf and Simmons 2009; Hoffmann et al. 2019). To our current knowledge, although there are analytical solutions dealing with a synthetic case of a cold water injection (Ascencio et al. 2014), this is the first time that cold water is used as an injected tracer in the field for hydrogeological aquifer characterization.

## Research Method

### Test Site

**Figure 1:**
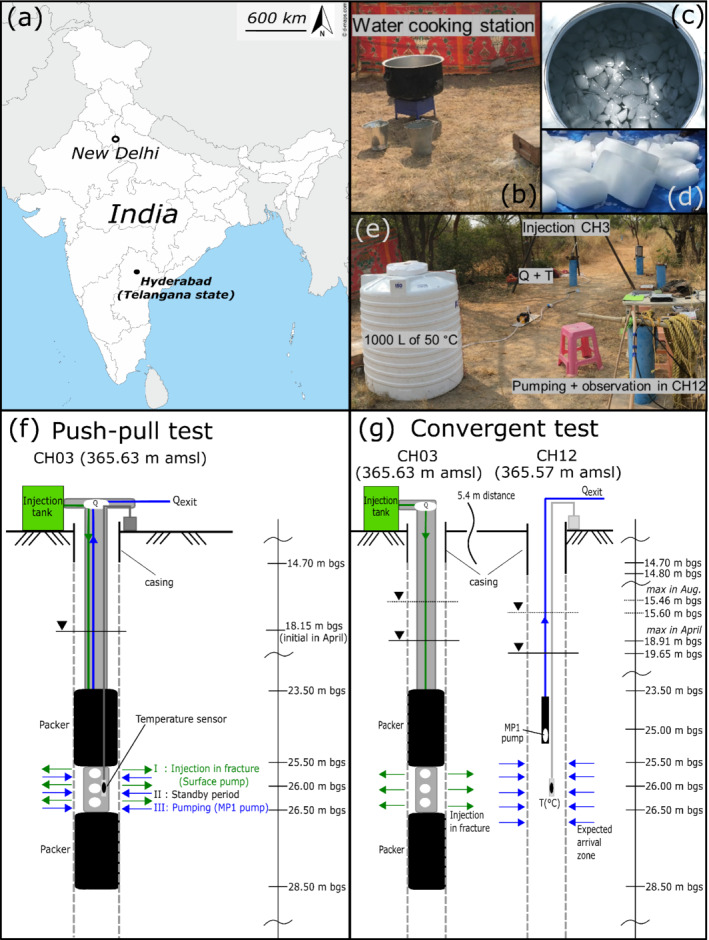
Test site location and experimental set‐up. (a) Location of the Environmental Hydrogeological Park (EHP) site in India. (b) Water heating system providing hot water for the injection fluid (50 °C). (c) Water ice blocks melting in the reservoir during preparation of the cold injection fluid (10 °C). (d) Water ice blocks prior to the experiment. (e) View of the injection (CH03) and the pumping (CH12) wells and the 1000 L reservoir exemplary for hot water injection (covergent test). (f) Push–pull experiment setup in well CH03. (g) Convergent experiment setup with injection in CH03 and recovery in CH12. The unit m bgs means “meters below ground surface.” The experimental design in (f) and (g) is comparable to the setup used by Guihéneuf et al. (2017).

The temperature tracer experiments were performed at the Experimental Hydrogeological Park (EHP) near the village of Choutuppal in southern India. The EHP is a scientific observatory for environmental research and is located around 60 km southeast of the state capital Hyderabad in Telangana state (Figure 1a). The French Geological Survey (BRGM) and the Indian National Geophysical Research Institute of Hyderabad (NGRI) are studying the impact of global (climate) and other local anthropogenic changes on the groundwater resources in a fractured weathered crystalline rock aquifer under high stress for irrigation (Maréchal et al. 2018). In the region of the EHP, the climate is semi‐arid and controlled by the periodicity of monsoons (Nicolas et al. 2019). Regional observations during the last decade show that the natural background groundwater temperature fluctuates by ±1 °C around 30 °C during the year.

A total of 30 boreholes were drilled at the EHP. These boreholes first intersect a deep saprolite (i.e., weathered granite bedrock) zone, which varies in thickness from 14 m to 24 m (Nicolas et al. 2019), and then the fractured Archean granite bedrock, which is characterized by an effective porosity less than 1% (Dewandel et al. 2006; Guihéneuf et al. 2014, 2017; Boisson et al. 2015). The experiments described in this study were performed between wells CH03 and CH12, which are 5.4 m apart. Well CH03 is around 50 m deep and CH12 is 56 m deep. Both wells are cased between the ground surface and the interface between the saprolite and the granite, to a depth of 14.70 and 14.80 m bgs (meters below ground surface), respectively.

**Figure 2:**
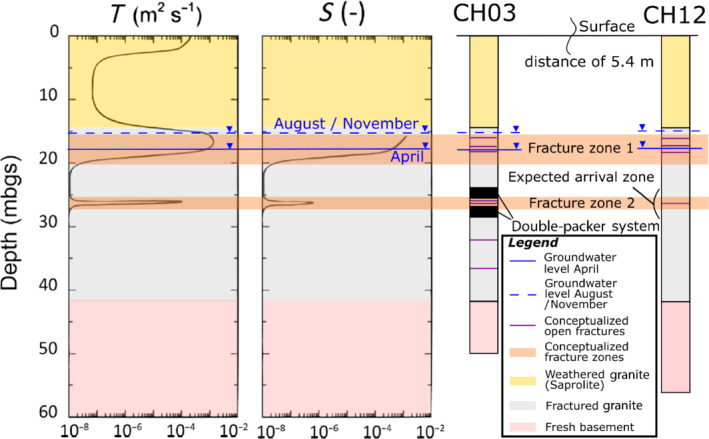
Typical transmissivity and storage profiles by depth from borehole CH03 are shown on the left side. These profiles are from Boisson et al. (2015) and have been modified by adding the horizontal units profile with depth, the depth to the natural groundwater level observed in November 2018, April 2019, and August 2019, and the identified fracture zones 1 and 2. Conceptual well profiles are added on the right side of the figure. These are derived from well logs and camera logging.

Wells CH03 and CH12 were previously used for pumping and tracer tests with injection of dye tracers (Guihéneuf et al. 2014, 2017; Boisson et al. 2015). Based on these tests and on well logs and camera images, two sub‐horizontally orientated fracture zones are considered in the current local conceptualization of the aquifer. They have an estimated lateral extent of tens of meters and they connect wells CH03 and CH12 (Guihéneuf et al. 2014, 2017; Boisson et al. 2015; Dewandel et al. 2018; Nicolas et al. 2019) (Figure 2). The first fracture zone is located at the level of the saprolite–granite interface, at about 14 m deep and consists of three closely spaced fractures whose saturation varies during the year (Nicolas et al. 2019) (Figure 2). The second fracture zone is located 26 m deep and remains fully saturated all year (Nicolas et al. 2019).

### Experiments

One push–pull experiment in well CH03 (Figure 1f) and five injection tests in CH03 with recovery in CH12 (i.e., forced gradient experiments in a convergent configuration; Figure 1g) were performed in November 2018, April 2019, and August 2019, under different hydraulic conditions (Table 1, Figure 2). The sub‐horizontal fracture zone located 26 m deep was isolated in the injection well CH03, using an inflatable double‐packer system with an open interval from 25.50 to 26.50 m bgs (Figure 1g and 1f). The hot and cold waters to be used for injection were prepared in situ in a plastic reservoir of 1000 L with foam insulation. The hot water was prepared using water heated with a gas cooker (Figure 1b). The cold water was prepared using water ice blocks submerged in the solution (Figure 1c and 1d). The temperature of the hot and cold injection waters was 50 °C and 10 °C, respectively (i.e., around 20 °C above and below the groundwater background temperature). During the experiment, the temperature of the water in the reservoir was measured and controlled continuously. A video showing the different steps of the experiments is available in Selles et al. (2019).

**Table 1:** Hydrogeological conditions during the heat tracer experiments with the measured natural groundwater depth in the injection (CH03) and extraction (CH12) wells.

		Natural groundwater depth below ground surface (m bgs)			Injection conditions (CH03)						Extraction(CH12)
Date	ID	CH03	CH12	Configuration	Tracer	Mass or Temperature	Volume (L)	Type	Time (h)	Flow rate (m^3^ h^−1^) (Surface Pump)	Flow rate (m^3^ h^−1^) (MP1 Pump)
November 2018	1	15.16	15.04	Convergent	Hot water	50 °C	975	Pulse	1	0.975	1^1^
					Salt	1 kg	10	Dirac	10 s	—	—
	2	15.16	15.04	Convergent	Cold water	10 °C	952	Pulse	1	0.952	1^1^
					Salt	1 kg	10	Dirac	10 s	—	—
April 2019	3	18.15	18.04	Convergent	Hot water	50 °C	952	Pulse	1	0.952	1
	4	18.15	18.04	Convergent	Cold water	10 °C	973	Pulse	1	0.973	1
	5	18.15	18.04	Push–pull	Cold water	10 °C	495	Pulse	0.5	0.991	1
August 2020	6	15.29	15.20	Convergent	Cold water	10 °C	986	Pulse	1	0.986	1

^1^ Flow rate in the extraction well was not constant.

The push–pull test, with injection of cold water in fracture zone 2 of well CH03, was performed in April 2019 (Figure 1f). This experiment was conducted in three successive steps. After injection of cold water for 30 min, a standby period with ambient flow conditions (no injection or withdrawal) of 30 min was maintained, and groundwater pumping was then activated for 23 h. Similar injection and pumping flow rates equal to about 1 m^3^ h^−1^ were operated by a surface and submerged pump, respectively (Table 1, Figure 1f). Temperature was monitored using a sensor located in the middle of the double‐packer chamber at a depth of about 26 m and thus within the open interval from 25.50 to 26.50 m bgs.

Convergent tests with injection of hot and cold water were performed in November 2018, in April 2019, and August 2019. Either hot or cold water was injected in CH03 within the inflatable double‐packer system for about 1 h and recovered in CH12 by pumping (Figure 1g). Table 1 summarizes the various experimental setups. To allow tracer comparison and assess the thermal retardation, 1 kg of salt dissolved in 10 L was simultaneously injected within the first few seconds of the hot and cold water injections performed in November 2018. The injection and pumping flow rates were equal to about 1 m^3^ h^−1^. The pump for tracer recovery was installed 25 m deep in CH12. Pumping in CH12 started significantly before the tracer injections to allow steady‐state conditions to develop. Temperature and salinity in CH12 were measured using sensors located at a depth of 26 m, and thus close to the openings of the fractures of fracture zone 2, which connect wells CH03 and CH12 and intersect well CH12 between 25.50 and 26.50 m bgs. In August 2019, the position of the sensor measuring temperature was checked with a borehole camera.

The natural groundwater level was different for each test. As listed in Table 1, all described experiments were carried out under different natural hydraulic conditions (Figure 2). In April 2019, the natural groundwater levels were about 18 m deep in the injection and extraction wells (Table 1). Considering this groundwater level, the deepest fracture of fracture zone 1 and the whole fracture zone 2 were saturated (Figure 2). In November 2018 and August 2019, the natural groundwater levels in the injection and extraction wells were about 15 m deep, inducing the full saturation of both fracture zones (Table 1, Figure 2). These different groundwater level conditions thus induce strong variations of the bulk aquifer transmissivity and storage coefficient, affecting fluxes and mixing conditions (Figure 2). Interpretation of the convergent tracer experiments must therefore be interpreted accordingly.

### Interpretation Method

The natural groundwater background concentration or temperature is first removed from the observed data using Equation (1):

(1) $$∆C\left(t\right)=C\left(t\right)-C\left(t_{0}\right)\;\text{or}∆T\left(t\right)=T\left(t\right)-T\left(t_{0}\right)$$

where ∆C(t) is the concentration difference (g L^−1^); C(t) is the measured concentration (g L^−1^); $C\left(t_{0}\right)$ is the background concentration (g L^−1^); ∆T(t) is the temperature difference (°C); T(t) is the measured temperature (°C); and $T\left(t_{0}\right)$ is the natural aquifer background temperature (°C).

The concentration differences ∆C(t) are expected to be always positive while the temperature differences ∆T(t) will be positive and negative for the hot and cold water injections, respectively. The data are then converted to a residence time distribution (RTD), assuming that all injections were performed as a pulse:

(2) $$p\left(t\right)=\frac{∆C\left(t\right)}{\int_{0}^{\infty }\left(∆C\left(t\right)\right)\cdot \mathrm{dt}}\;\text{or}\;p\left(t\right)=\frac{∆T\left(t\right)}{\int_{0}^{\infty }\left(∆T\left(t\right)\right)\cdot \mathrm{dt}}$$

where *p*(*t*) is the RTD function (s^−1^).

Equations (1) and (2) allow normalizing breakthrough curves to compare them in terms of peak time, peak value, and slope of the curve tails. The slopes of the breakthrough curve tails are characterized in log–log plots (Kang et al. 2015).

The convergent tests from April 2019 and August 2019 without salt injection are used for calculating the thermal recovery rate (de la Bernardie et al. 2019) and the cumulative energy recovery. Such measures of the total tracer recovery in a pumping well can indicate unforeseen losses due to the heterogenous nature of the actual transport processes which possibly include adsorption and/or degradation (Dassargues 2018). This provides useful information for the parameterizations determined or tested by inverse modeling techniques. During a thermal tracer test, the quantity of energy in the circulating fluid increases (hot water) or decreases (cold water). The thermal energy change $∆E_{\text{thermal}}\left(t\right)$ can then be calculated as:

(3) $$\begin{array}{cc} ∆E_{\text{thermal}}\left(t\right) & =m_{w}\cdot c_{w}\cdot ∆T\left(t\right)\\ & =V_{w}\cdot \rho_{w}\left(T\right)\cdot c_{w}\cdot ∆T\left(t\right)\\ & =\left[Q_{\text{flow}}\left(t\right)\cdot t\right]\cdot \left[\rho_{w}\left(T\right)\cdot c_{w}\right]\cdot ∆T\left(t\right) \end{array}$$

where $∆E_{\text{thermal}}\left(t\right)$ is the thermal energy change (J); $m_{w}$ is the tracer water mass (kg); $c_{w}$ is the water specific heat capacity (J kg^−1^ K^−1^); ∆T(t) is the temperature difference (Equation 1) (°C or K); $V_{w}$ is the water volume transporting the energy, i.e., injection volume (m^3^); $\rho_{w}\left(T\right)\cdot c_{w}=s$ is the water volumetric heat capacity (J K^−1^ m^−3^); *Q*_flow_(*t*) is the water flow rate (m^3^ s^−1^); *t* is the corresponding time (s); and $\rho_{w}\left(T\right)$ is the temperature‐dependent density function of the water (kg m^−3^).

Equation (3) can be used to express the thermal power, individually for the injection and pumping points, by dividing the thermal energy change by the corresponding injection and observation time, respectively. For fractured rocks, the thermal recovery rate *r*_thermal_ (*t*) expresses the relation between the injection power *P*_in_ and the instantaneous recovery power *P*(*t*) (Equation 4) (de la Bernardie et al. 2019):

(4) $$\begin{array}{cc} r_{\text{thermal}}\left(t\right) & =\frac{P\left(t\right)}{P_{\text{in}}}\\ & =\frac{\left(\frac{∆E_{\text{thermal}}\left(t\right)}{t}\right)}{\left(\frac{∆E_{\text{injected}}}{t_{\text{injection}}}\right)}\\ & =\frac{c_{w}\rho_{w}\left(T\right)\;Q_{\text{outflow}}}{c_{w}\rho_{w}\left(T\right)\;Q_{\text{inflow}}}\cdot \frac{∆T\left(t\right)}{\left(T_{\text{injection}}-T_{\text{Background}}\right)} \end{array}$$

where *P*(*t*) is the instantaneous recovery power (W); *P*_in_ is the injection power (W); *Q*_inflow_ is the water injection flow rate (m^3^ s^−1^); *Q*_outflow_ is the water extraction flow rate (m^3^ s^−1^); *T*_injection_ is the temperature of the injected water (°C); and ∆T(t) is the temperature difference (Equation 1) (°C or K).

Considering temperature differences, complete mixing of the tracer, and constant flow rates, the cumulative energy recovery can be expressed as follows:

(5) $$r_{\text{energy}}\left(t\right)=\frac{\left[c_{w}\rho_{w}\left(T\right)\cdot Q_{\text{outflow}}\cdot \int_{0}^{t}\left(∆T\left(t\right)\right)\cdot \mathrm{dt}\right]}{∆E_{\text{thermal}}^{\text{injected}}}$$

where $r_{\text{energy}}\left(t\right)$ is the relative energy recovery at time *t* (−); ∆T(t) is the temperature difference (Equation 1) (°C or K); and $∆E_{\text{thermal}}^{\text{injected}}$ is the injected thermal energy difference (Equation 4) (J).

Both kinds of recovery are tested to quantify the tracer recovery of hot and cold water injections (i.e., heat tracer experiments) in a similar way as usually done for solute mass tracers. In addition, recovery values have also been used to compare the effects of hot and cold water injections on the direction of thermal conduction.

Following these first interpretations, experiment results of the convergent tests performed in April 2019 and August 2019 are then simulated numerically, using the finite‐element code HydroGeoSphere (HGS) (Therrien et al. 2010) using the control volume approach with a full three‐dimensional (3D) model. The fracture zones shown in Figure 2 are represented as two‐dimensional (2D) planes (discrete fractures) embedded into a 3D porous medium. Such a simplified numerical model used to describe the experimental observations is useful for obtaining a parameterization by calibration or inverse modeling. Then, it allows to first understand how parameter values change (e.g., average fracture aperture) for the different processes considered to influence the results (e.g., positive and negative evolutions in temperature as a function of time). The model will provide information on the impact of heat conduction toward and from the porous rock matrix on groundwater heat transport in the weathered and fractured granite at the meter scale.

## Tracer Test Results

### Push–Pull in Well CH03

**Figure 3:**
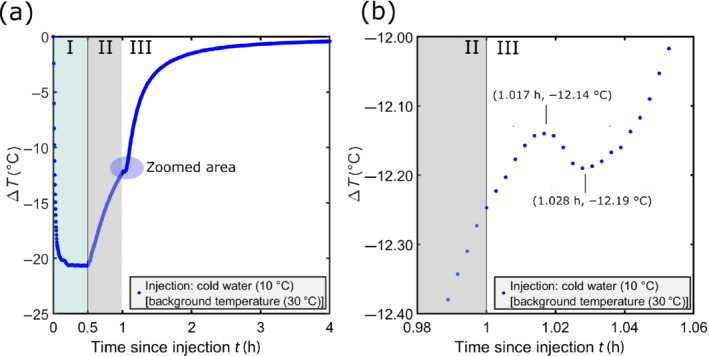
Measured temperature in well CH03 during the push–pull experiment with cold water injection. The experiment consists of three phases: I: injection (30 min); II: standby (30 min); and III: pumping (23 h). (a) Temperature difference signal observed in CH03. (b) Zoomed area (blue circle in Figure 3a) to visualize the observed temperature rebound, between the standby period (II) and the pumping period (III). The increase of temperature observed during the standby stage is temporarily stopped at the beginning of the pumping stage, before increasing again at a higher rate.

Results of the push–pull test performed in April 2019 are shown in Figure 3. After 12 min of cold water injection, that is, when around 20% of the water volume has been injected, a stabilized temperature anomaly of ∆*T* = −20.69 °C was measured in the middle of the injection chamber for the rest of the injection phase (I) (Figure 3a). The measured absolute temperature of around 10 °C is similar to the water temperature in the surface tracer tank (Experiments section). During the standby period (II), the temperature immediately increases toward natural background values. The increase is temporarily interrupted at a temperature difference of −12.14 °C, at the beginning of the pumping stage (phase III—see Figure 3). The temperature then increases again at a higher rate. After this rebound, the temperature starts again to increase toward the natural background groundwater temperature.

### Convergent Tests

**Figure 4:**
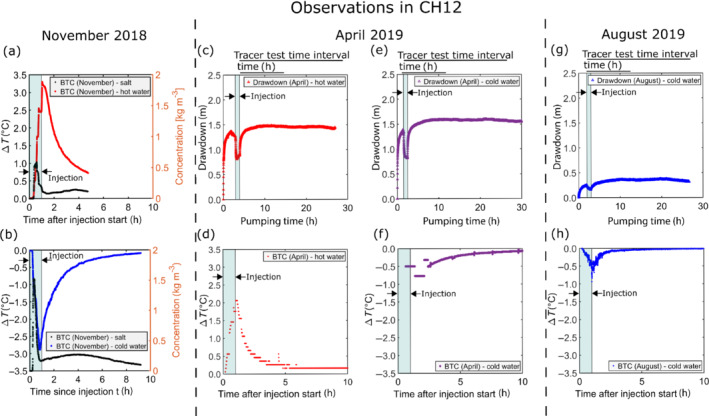
Tracer test observations in convergent configuration for (a, b) November 2018, (c–f) April 2019, and (g and h) August 2019. Results for November are shown for an injection in well CH03 of hot water (50 °C) (a) and cold water (10 °C) (b) complemented by a salt injection performed within a 10‐s interval at the beginning. Figures (c, e, and g) show the observed drawdown in the pumping well CH12 and figures (d, f, and h) show a zoom of the temperature evolution as a function of time (BTC: breakthrough curve) measured in the pumping well CH12 for a (c, d) hot water injection in April 2019 (50 °C), (e,f) cold water injection (10 °C) in April 2019, and (g, h) cold water injection in August 2019. (Note that no information about the flow rate (Q‐t‐s‐diagram) is given, because the flow rate was measured manually, and no pumping test analysis was performed. The drawdown values for November are not mentioned as the pump stopped accidentally several times). Stationary drawdowns of 1.45 m, 1.55 m, and 0.35 m were measured for the hot water injection in April, the cold water injection in April, and the cold water injection in August, respectively. The corresponding maximum temperature differences were +2 °C and −1 °C for the hot water injection in April, and the cold water injection in August.

Results of the five convergent tests are shown in Figure 4.

In November 2018, the extraction pump in CH12 stopped accidentally several times, inducing unwanted steps in the signal of Figure 4a. Nevertheless, observed peak times for the hot and cold water injections in November 2018 are around 1 h after the injection started and the peak temperature changes ∆*T* are about +3.4 °C and −2.9 °C, respectively. In contrast, the observed peak times for the salt tracer, injected jointly with the hot and cold water in November 2018, are 0.58 h and 0.34 h, respectively, while the peak changes in the concentration ∆*C* of the salt tracer are about 0.5 kg m^−3^ and 1.5 kg m^−3^, respectively. The longer peak time and the smaller peak change in concentration of the salt tracer, when hot water is injected, is mainly related to the intermittent stops of the extraction pump.

In April and August 2019, pumping in well CH12 was started significantly before the injections in CH3. Steady‐state conditions were reached after a few hours, but injections were performed before reaching steady‐state sensu stricto. During the injection periods, the groundwater drawdown was temporarily decreased. In April 2019, stationary drawdowns of 1.45 m and 1.55 m were measured in the pumping well, for the hot and cold water experiments, respectively (Figure 4c and 4e). The observed drawdown was higher for the cold water injection. This difference may be attributed to higher groundwater viscosity and density, induced by lower temperatures and considering a constant pumping flow rate. In August 2019, the stabilized drawdown in CH12 was significantly lower and equal to 0.35 m (Figure 4g). This clearly shows different hydrogeological conditions and the strong influence of the fractures located around the saprolite–granite interface (Fracture zone 1; Figure 2), all of them being fully saturated in August 2019. The pumping flow rate in CH12 was thus distributed among more fractures in August 2019, inducing lower hydraulic gradients and lower groundwater fluxes in the fracture investigated by the tracer experiments.

Temperature breakthrough curves are shown in Figure 4d, 4f, and 4h. Records in well CH12 are characterized by different temperature resolutions, making the estimation of the peak time and value difficult for the cold water injection in April 2019 (Figure 4f). Observed peak times for the hot and cold water injections are around 1 h after the injection started. The peak temperature changes ∆*T* are about +2 °C and −1 °C, respectively, for the hot water injection in April and cold water injection in August, when the hydraulic situation has changed.

### Analysis of Breakthrough Curve Tails

**Figure 5:**
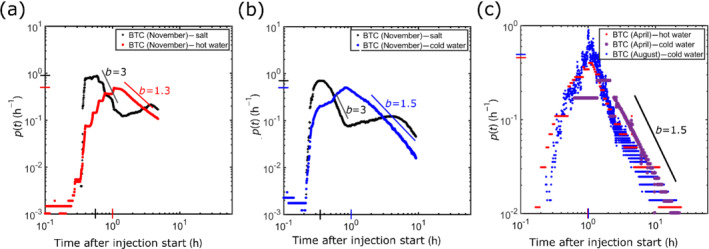
Tracer observations of the convergent tests (BTC: breakthrough curve) converted to residence time distribution. For November 2018 (a) joint hot water (50 °C) and salt injection and (b) joint cold water (10 °C) and salt injection. Figure (c) shows the hot (50 °C) and cold (10 °C) water injection in April 2019 and the cold water (10 °C) injection in August 2019. Dimensionless slopes of about 1.5 and 3.0 were estimated for the thermal tracers and the salt tracer, respectively. The smaller slope for the temperature tracers is explained by a stronger thermal diffusive behavior, while the less diffusive salt tracer is mainly advective transported.

Figure 5 shows the convergent test results converted to residence time distribution *p*(*t*) (Equation 2), for comparison of the peak times as well as peak and tail slope values, given the different injection functions. Results of the two joint “salt‐heat” tracer tests (November 2018) allow a comparison of the simultaneous salt and heat transfer processes. Similar first arrival times are observed for the salt, cold water, and warm water tracer fluids (Figure 5a and 5b). The peak arrival times related to salt are significantly shorter than those observed for hot (Figure 5a) and cold (Figure 5b) water injections. This is consistent with thermal retardation induced by fracture‐matrix exchanges, which delays transport times. The measured residence time *p*(*t*) peak values for salt, injected jointly with the hot and cold water, are 0.9 h^−1^ and 0.7 h^−1^, respectively. In contrast, the peak values of all temperature breakthrough curves in the residence time distribution are close to 0.5 h^−1^ (Figure 5).

The residence time distribution functions visualized in log–log format show breakthrough tails as straight lines (Kang et al. 2015). The tail slope values of the salt tracer tests are estimated to be around 3. Observed tail slopes of all hot and cold water breakthrough curves are close to 1.5, corresponding to a stronger thermal diffusive behavior, explained mostly by heat conduction (Kang et al. 2015; Hyman et al. 2019; Hoffmann et al. 2020). This clear difference between the slopes observed for the salt and temperature records indicates the complementary nature of both types of experiments to be used for differentiating fracture and matrix characteristics and especially the impact of diffusive and conductive processes on solute and heat transport.

### Thermal Recovery

**Figure 6:**
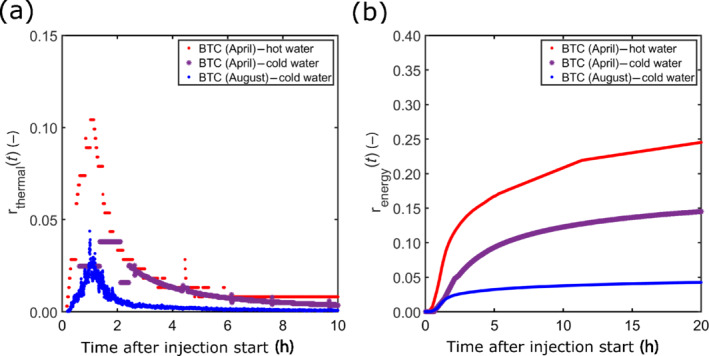
(a) Estimated thermal recovery rate and (b) cumulative energy recovery for the convergent experiments (BTC: breakthrough curve) performed in April and August 2019. Both tend to be smaller for a cold water injection, which is related to a possible density‐viscosity effect.

Figure 6 shows the instantaneous thermal recovery rate and the cumulative energy recovery of the convergent tests of April and August 2019 (Equations 4 and 5). The convergent tests of November 2018 are not considered here as the extraction pump in CH12 stopped accidentally several times. The peak of the thermal recovery rate for the hot water injection in April and the cold water injection in August is equal to about 11% and 2.5%, respectively (Figure 6a). Twenty‐five hours after the beginning of the injection, the cumulative energy recovery for the hot water injection in April and the cold water injection in August is equal to 26% and 4.5% (Figure 6b). The rough estimation of the thermal recovery rate and the cumulative energy recovery for the cold water injection in April 2019 leads to intermediate results. Values are lower compared to the hot water injection under similar hydrogeological conditions, but higher than for the cold water injection in August 2019. The lower recovery rates for August 2019 may be related to the different hydrogeological conditions, including more saturated fractures in fracture zone 1, resulting in lower groundwater flux values in the investigated fracture and more dilution in the extraction well. Results are evaluated further using process‐based numerical modeling of the observed system conditions, described in the next section.

## Modeling

### Model Setup

**Figure 7:**
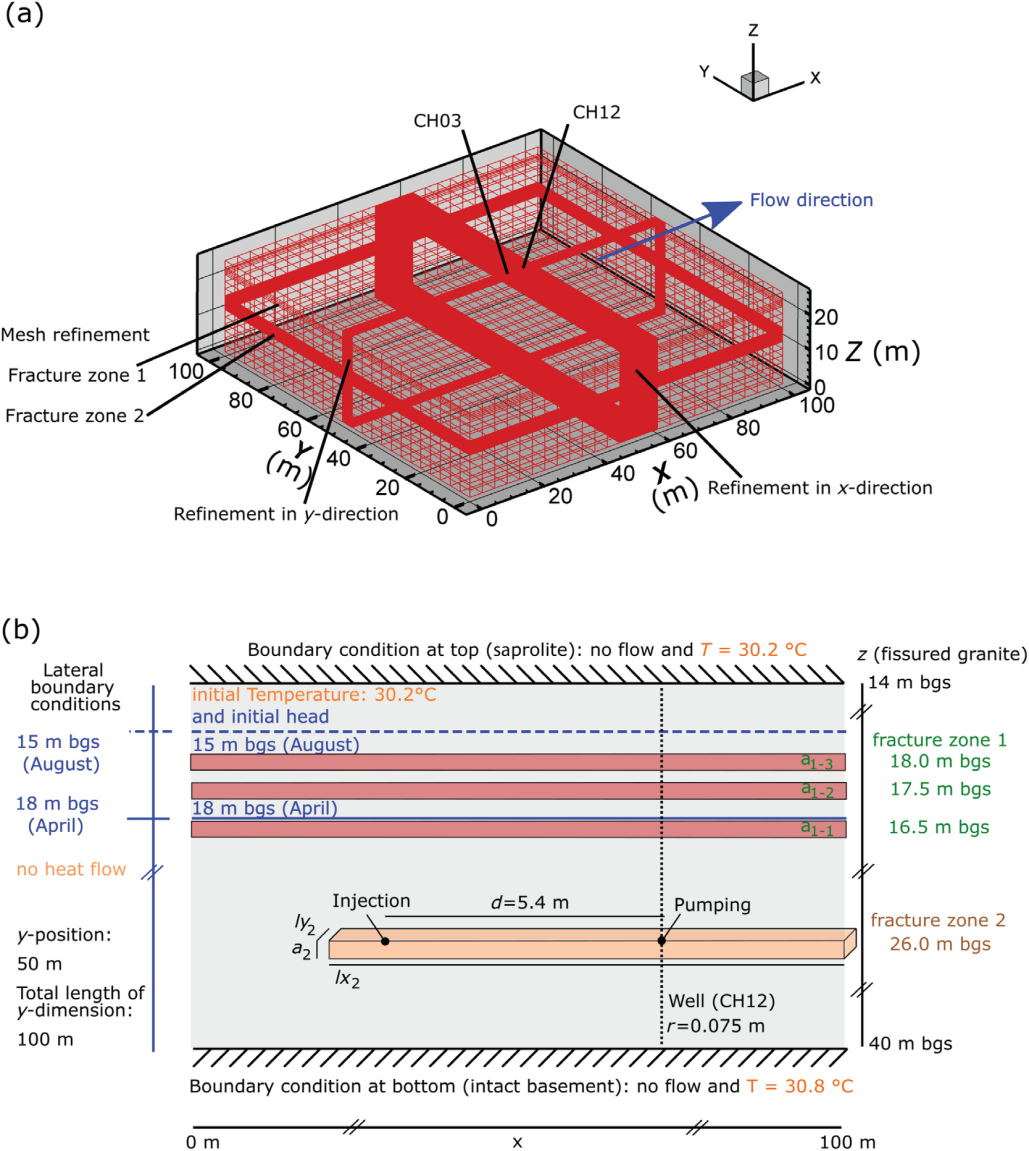
Explanation of the model discretization. (a) The mesh with the refinement sections. (b) Schematic cross‐section of the vertical plane at the position y = 50 m of the 3D numerical model. Note, “m bgs” stands for meters below ground surface. A discretely fractured porous media is modeled with HydroGeoSphere.

Results from the convergent temperature tracer tests of April and August 2019, with constant pumping rates, are interpreted using a 3D numerical model using HGS (Therrien et al. 2010; Klepikova et al. 2016b). Groundwater flow and heat transport are simulated for discretely fractured porous media. The model considers density‐viscosity dependent flow to account for dynamic changes of hydraulic parameters due to changes in groundwater temperature when injecting hot and cold water. The dimensions of the model grid are 100 m × 100 m × 26 m, covering the thickness of the fractured granite measured at the wells CH03 and CH12 (Figures 2 and 7). Grid cells are hexahedral blocks with a lateral extension of 0.2 × 0.2 m in the area of the two wells (*x* = [48, 60] and *y* = [48, 52] m), and 4.0 m × 4.0 m elsewhere. Outside the fracture zones, the cell thickness is 2 m. In fracture zones 1 and 2, from *z* = 16 to 18 m bgs and 24 to 28 m bgs, the mesh is vertically refined by a factor of 4 and 10, respectively (Figure 7a). In accordance with the conceptualization of the site (Figure 2), three horizontal discrete fractures, located 18.0, 17.5, and 16.5 m bgs are implemented within fracture zone 1, over the whole model domain (Figure 7b). One horizontal discrete fracture, located 26 m bgs, is implemented within fracture zone 2. The thickness of each fracture (a_1‐1_, a_1‐2_, a_1‐3_ in fracture zone 1 and a_2_ in fracture zone 2) and the lateral extension of fracture zone 2 (*lx*_2_ × *ly*_2_) are adjustable parameters (Figure 7b) of the model. The extraction well CH12 is implemented as a polyline element over the full aquifer thickness, representing a tube of 0.075 m radius (Figure 7b). The intersections of this polyline with the fractures allow the representation and calculation of the water flows from the discrete fractures to the well at different depths (Therrien et al. 2010; Dewandel et al. 2018). The model grid includes 137,268 elements and 140,680 nodes.

A fixed hydraulic head boundary condition is defined along the external lateral boundaries of the grid, far from the injection and extraction wells (Figure 7b). Prescribed values correspond to the hydraulic heads in April and August (18 and 15 m deep, respectively) and account for the different numbers of saturated fractures. Note that in April, when groundwater levels are lower, the two uppermost fractures of fracture zone 1 are desaturated. No‐flow boundary conditions are implemented at the top and bottom of the model, assuming no flow exchange from the granite basement and neglecting groundwater recharge fluxes at the time scale of the experiments. Neglecting recharge may be a simplification for August (monsoon season) but is considered to not have influenced the experiment, since realistic groundwater level conditions are implemented according to the period. Fixed temperature values of 30.2 °C and 30.8 °C are defined at the grid top and bottom, respectively, according to the local geothermal gradient (Figure 7b). Flow and temperature initial conditions correspond to a steady‐state run, without any pumping or injection in the wells.

In the extraction well CH12, a flow rate of −2.5 × 10^−4^ m^3^ s^−1^ is prescribed for all simulations (Figure 7b). HGS calculates fluxes coming from the different medium sections into the well, as a function of the implemented hydraulic conditions (Therrien et al. 2010). This allows properly considering the different hydrogeological conditions during the April and August experiments, with the activation or deactivation of the upper fractures in fracture zone 1. Pumping operations are started before injection, in accordance with experimental conditions. The injection is defined at a specific node located 5.4 m from the extraction well, in fracture zone 2. Prescribed flow rates are 2.5 × 10^−4^ m^3^ s^−1^ and 3.0 × 10^−4^ m^3^ s^−1^ during 1 h for the April and August experiments, respectively (Figure 7b). Temperature of the injected water is prescribed according to the experimental conditions (Table 2).

**Table 2:** Chosen parameter values for the granite system (left column) and adjusted parameter values for the 3D matrix and 2d fracture plane (right column)

Fixed parameters			Calibrated parameters		
Name	Value	Unit	Name	Value	Unit
Thermal properties			Porous medium		
Thermal conductivity of water	0.59	W m^−1^ K^−1^	Effective transport porosity	0.5	%
Specific heat capacity of water	4189	J kg^−1^ K^−1^	Hydraulic conductivity	10^−9^	m s^−1^
Specific heat capacity of the solid	780	J kg^−1^ K^−1^	Specific storage coefficient	3.0 × 10^−3^	m^−1^
Thermal conductivity of solid	3.50	W m^−1^ K^−1^			
Porous medium			Fracture zone 1 (saprolite–granite)		
Dry bulk density	2750	kg m^−3^	Aperture a_1‐1_ (saturated in April and August 2019)	0.58	mm
Dispersivity	0	m	Aperture a_1‐2_ and a_1‐3_ (saturated only in August 2019)	0.80	mm
			Hydraulic conductivity (computed)		
			For aperture a_1‐1_	0.24	m s^−1^
			For aperture a_1‐2_ and a_1‐3_	0.46	m s^−1^
			Storage coefficient (computed)		
			For aperture a_1‐1_	4.3 × 10^−6^	
			For aperture a_1‐2_ and a_1‐3_	4.3 × 10^−6^	
			Fracture size (*lx*_1_, *ly*_1_)	100, 100	m
			Fracture zone 2		
			Aperture a_2_	0.75	mm
			Hydraulic conductivity (computed)	0.41	m s^−1^
			Storage coefficient (computed)	4.3 × 10^−6^	—
			Fracture size (*lx*_2_, *ly*_2_)	50.6, 1	m

Notes: The stresses are an extraction flow rate of −2.5 × 10^−4^ m^3^ s^−1^, and an injection flow rate for April and August 2019 of 2.5 × 10^−4^ and 3.0 × 10^−4^ m^3^ s^−1^, respectively.

### Parameterization

Table 2 summarizes the fixed and adjustable parameters of the model. The aquifer is represented as a low‐porosity and low‐hydraulic‐conductivity medium, intersected by highly transmissive fractures. Standard fixed values are used for the dry bulk density of the fractured granite as well as for the thermal conductivity and specific heat capacity of the water and fractured granite (Table 2). The heat dispersivity in the porous medium is neglected. Sensitivity to this parameter is nevertheless very low in a porous medium with limited advection and dominated by thermal conduction (Anderson 2005; Rau et al. 2012; Dassargues 2018).

The hydraulic conductivity, specific storage coefficient and effective transport porosity of the porous media, as well as the apertures of the discrete fractures, are implemented as adjustable parameters.

### Calibration

**Figure 8:**
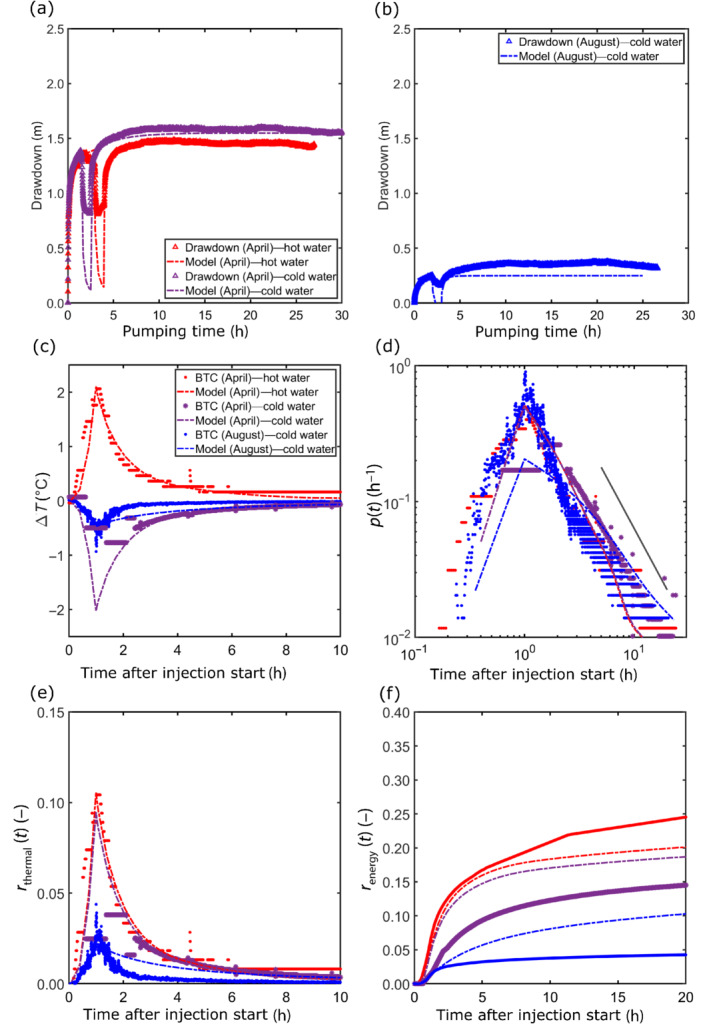
Observations in CH12 compared with the model. Observed and simulated drawdown (a) for the hot (50 °C) and cold (10 °C) water injection in April 2019 and (b) the cold water (10 °C) injection in August 2019. Temperature observations (BTC: breakthrough curve) and simulations for the hot (50 °C) and cold (10 °C) water injection in April 2019 and the cold water (10 °C) injection in August 2019 (c) presented as temperature difference and (d) converted to residence time distribution (Equation 2) and visualized in a log–log format. Observed and simulated thermal recovery rate (e) and energy recovery (f) for the hot (50 °C) and cold (10 °C) water injections in April 2019 and the cold water (10 °C) injection in August 2019. Legend for figures (c) to (f) is shown in figure (c). The simulated stationary drawdown magnitudes are 1.6 m, 1.4 m, and 0.4 m for the hot water injection in April, the cold water injection in April, and the cold water injection in August, respectively. The simulated temperature values are also close to the observed temperatures.

The adjustable parameters are manually calibrated. Residuals between the observed and simulated drawdown and temperature in CH12 are minimized simultaneously and with equal weight (Figure 8). The experiments of April and August are complementary, as hydrogeological conditions are different. Results in April bring information for calibrating flow parameters of the rock matrix, fracture zone 2, and the deepest fracture of fracture zone 1. Results in August provide complementary data to calibrate the flow parameters of the two uppermost fractures, which are saturated during this period (Figures 2 and 7).

**Figure 9:**
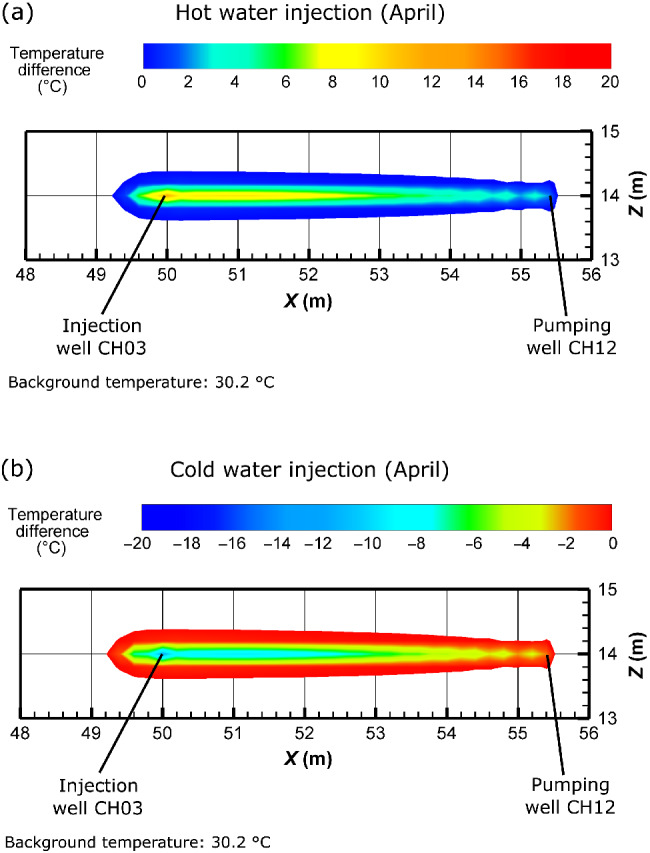
Simulated temperature distributions as differences corresponding to the hot (50 °C) and cold (10 °C) water tracer experiments performed in April 2019, at the end of the injection period. Zoom in the vertical plane for (a) the hot water and (b) the cold water injections. The extensions of the impacted areas are similar.

Calibrated parameter values are presented in Table 2. Equivalent hydraulic conductivity and storage coefficient values are calculated for each of the four fractures, based on their calibrated aperture and using the cubic law (Therrien et al. 2010). Corresponding simulations compared to observed data are shown in Figure 8. Examples of the simulated temperature spatial distribution are presented in Figure 9. Calibrated parameter values of the granitic porous medium logically correspond to a low‐permeability low‐porosity medium. The calibrated apertures of the unique fracture of zone 2 (a_2_) and the three fractures of zone 1 (a_1‐1_, a_1‐2_, and a_1‐3_) are included between 0.58 mm and 0.80 mm. As conceptually considered and in agreement with Guihéneuf et al. (2014) the extension of fracture zone 2 is limited. While the calibrated length in the flow direction (*lx*_2_) is 50.6 m, which allows connection between the two wells, the width of the fracture (*ly*_2_) is equal to only 1 m (Figure 7b). In addition to the aperture influencing flow and transport simulations, the fracture width shows a high sensitivity during the manual calibration and allows a good calibration of the temperature breakthrough curves and a correct thermal recovery rate.

Simulations of the three experiments based on the calibrated parameters consider the actual hydraulic conditions. In April, consistently with the observations (Figure 8a), the model simulates a slightly higher drawdown when cold water was injected. The maximum simulated drawdowns are 1.6 m and 1.4 m, respectively, for the hot and cold water injections in April (Figure 8a). The maximum drawdown simulated in August is 0.4 m (Figure 8b) and thus smaller compared to April, which is consistent with the lower transmissivity in April. This confirms the effect of density and viscosity changes due to temperature variations, as discussed earlier. In contrast, in August when two more fractures are saturated, the model drawdown is significantly underestimated compared to measured data after 4 h (Figure 8b). The most notable comparison (mismatch of model to data) is during the injection. The model consistently overestimates the observed drawdown reduction during injection periods (Figure 8a and 8b), which may arise from a lack of information about vertical fractures and interconnectivity between the fracture zones. Regarding the temperature simulations in CH12, the measured peak and tailing of temperature changes for the hot water injection in April is well simulated (Figure 8c). In contrast, for the cold water injection in August, the measured smaller peak linked to the changed hydraulic condition is also well simulated but the tailing is overestimated (Figure 8c). The observed slopes of 1.5 are roughly reproduced for each thermal tracer test (Figure 8d).

Regarding the thermal recovery rate and the cumulative energy recovery, the model results confirm the lower values when cold water is injected (Figure 8e and 8f). The only differences between the simulations of the hot and cold water injections performed in April are actually the inverted temperature anomaly and the pumping time before injection. Thus, identical parameter values were used. For the hot water injection, the direction of heat transfer is mostly “from fracture to matrix” and for the cold water injection this is “from matrix to fracture.” The simulated drawdown before injection is almost similar for a hot and a cold water injection in April, while the stationary drawdown magnitude after a cold water injection is 0.2 m higher than after a hot water injection (Figure 8a and 8c). The vertical extension of the impacted area (temperature difference) is shown in Figure 9, at the end of the injection of hot and cold water in April, respectively. The simulated temperature distributions are relatively similar, and no significant difference is visible when comparing the hot and cold plume (Figure 9). One might expect the size of a hot and cold plume should be somewhat different, since the values for thermal conductivity and specific heat capacity of water and solids also change as a function of the fluid temperature. We have not considered this effect, because hot water injections have been plausibly simulated so far when constant values of thermal conductivity and specific heat capacity of water and solid were used (e.g., Hermans et al. 2018; Hoffmann et al. 2019). However, it may be worthwhile to consider all thermal parameters as temperature dependent (as a function of the fluid temperature) when simulating heat transport, since possible different distributions of the hot and cold water plumes along the fracture network pathways could possibly be observed.

## Discussion

For this weathered and fractured granite aquifer in India with a natural background temperature of around 30 °C, it was indeed interesting to inject cold water (i.e., injection temperature < groundwater temperature) and to compare with hot water (i.e., injection temperature > groundwater temperature) and salt tracer tests. The interpretation and deterministic transport modeling of multiple temperature tracer experiments performed in different hydrogeological conditions have provided useful information about heat transport “from fracture to matrix” and “from matrix to fracture.” The convergent tests with heat and salt allowed a comparison of the breakthrough curve peak arrival times, showing that the hot and cold water arrivals are delayed compared to the salt tracer. On the other hand, the delay due to heat losses to zones of low hydraulic conductivity and to the solid matrix (“thermal retardation”) seems short. This fast energy exchange between fracture and matrix is supported by the results of a push–pull experiment showing that energy is not stored in the matrix for a very long time and the amount of heat back‐released to the circulating groundwater is small. This low retardation was expected compared to observations of a hot water injection in a highly porous fractured chalk aquifer (Hoffmann et al. 2021). Nevertheless, as hot water, cold water, and salt tracers affect the density of groundwater differently, salt is clearly not the best tracer to be injected simultaneously with a thermal tracer. Thus, if possible, a dye tracer should be preferred, which was here technically not possible. However, the slope of the tailing of all breakthrough curves observed during the convergent tests can be analyzed. The slopes for the salt tracer were around 3.0 while those for the thermal tracers were around 1.5 and independent of the hydrogeological conditions. A second salt peak was observed that is significantly lower than the first peak, similar to results from Guihéneuf et al. (2017), who injected a fluorescein tracer and simulated solute transport with a multi‐path analytical solution. Salt has a molecular diffusion coefficient of about 10^−10^ m s^−1^ and is thus mostly transported by advection and mechanical dispersion. In this context salt tends to be transported farther in convergent tracer tests compared to tracers with a stronger diffusive behavior, whose interactions with the matrix are intensified (Hoffmann et al. 2020). The second peak is therefore mostly caused by tracer mass pushed upgradient, which arrives with delay either due to a longer travel distance in the same fracture or by traveling through a second fracture, which was simulated by Guihéneuf et al. (2017). The double peak was not observed after the injection of hot and cold water, because heat is also transported by conduction through the pores/fractures and the solid matrix, and by advection/convection through the fractures. This causes some potential heat losses toward the solid or some heat release from the hotter domains to the colder circulating fluid in the fractures. The result is a longer breakthrough curve tail and a smaller slope, as observed here for the granite.

The higher slope for salt in the present study, compared to the slopes of uranine in Guihéneuf et al. (2017), is probably associated with a density effect, but still represents advection‐dominated transport. For heat transport, the heating and cooling of the matrix is controlled by conduction that plays an important role. The slopes of 1.5 estimated for the thermal tracers clearly highlight this effect of heat conduction and heat storage (Kang et al. 2015; Hyman et al. 2019; Hoffmann et al. 2020).

The convergent tests performed in April and August 2019 with injection of hot and cold water were simulated using HGS. The adopted conceptual model is relatively simple in terms of fracture properties (no roughness, average apertures) and saturation, and thus calibrated parameter values are only indicative values. A reasonably good fit was obtained for the simulation of the hot and cold water breakthrough curves considering, respectively, the groundwater levels in April (Indian summer) and August (Monsoon period). The horizontal dimensions (*x*‐ and *y*‐direction) of fracture zone 2 (26 m deep and always saturated), were adjusted. This adjustment can be understood in a way that heat transport in a porous medium with a low hydraulic conductivity is controlled by conduction, while heterogeneous advection controls the transfer times in the embedded fractures. Heat and (conservative) solute transport are not influenced by the same processes. Solute transport is affected by advection, hydrodynamic dispersion, and matrix diffusion caused by concentration differences between mobile and immobile water. Heat transport is affected by advection/convection, mechanical dispersion, and by diffusion including mostly conduction in water and in the solid. Heat transport is retarded compared to the transport of a conservative solute, due to the heat conduction in the solid. The effect of conduction is confirmed by tail slopes of about 1.5 for the thermal tests and tail slopes of 3 for a tracer with a weaker diffusive behavior (salt). Slopes are smaller, when matrix interactions are intensified (Kang et al. 2015).

The determined parameters in this study should be considered as only one possibility to describe reality and should be used with caution for predictions. For example, no vertical fracture information is considered, as no reliable field information is available yet. The lack of vertical fractures in our conceptual model (Figure 2) could be the main reason why the observed drawdowns during the injection periods in April and in August were not well simulated by the model.

The transport model allows a first discussion on the influence of the different heat transport process directions on the breakthrough curves. Advective transport of the hot and cold water cases occurs mainly in the fractures and to a lesser extent in the porous matrix. In contrast, conduction always occurs in the direction of the negative temperature gradient. When hot water is injected in the fracture, the heat conduction direction is mainly from the hot circulating fluid in the fracture toward the colder porous matrix. Heat is thus mainly transferred from the fracture, which is considered as a continuum with high specific heat capacity, low thermal conductivity, and high hydraulic conductivity, into the porous matrix, which is considered as a second continuum with low specific heat capacity, high thermal conductivity, and low hydraulic conductivity. In addition, later, it is possible that heat is transferred back from the porous medium to the fracture during the tailing period. In comparison, the heat conduction direction is inverted for a cold water injection. Heat is transferred directly from the porous medium to the colder circulating groundwater in the fractures during the entire experiment. Observations and simulations for the same hydrogeological conditions (i.e., April) have shown that thermal recovery rates tend to be lower for a cold water injection than for a hot water injection. This is consistent with the expectation, that a hot plume should render the system more permeable allowing more advective transport, due to a density‐viscosity effect. In addition, we speculate that the cold water plume is more strongly influenced by fracture roughness and is thus unevenly distributed along the fracture plane, limiting the surface area for conduction compared to the lower viscosity hot water condition. A detailed evaluation of the critical Reynolds number as a function of the aperture (e.g., Quinn et al. 2020) as well as a sensitivity analysis while also considering the thermal conductivity and specific heat capacity values as dependent on the fluid temperature may provide new insights, but this is beyond the scope of this study.

## Conclusions

In this study hot (50 °C) and cold water (10 °C) were injected into a fractured granite aquifer with a natural groundwater background temperature of 30 °C. A permanently saturated fracture was isolated using an inflatable double‐packer system and the temperature evolution as a function of time was observed during a push–pull test in the injection well (CH03) and five additional convergent tests were performed between two wells (CH03 and CH12) separated by 5.4 m. The experiments were performed in November 2018, April 2019 and November 2019. The depth to the natural groundwater level was 3 m lower in April 2019 than in November 2018 and August 2019 inducing different hydrogeological conditions. Observed slopes of the breakthrough curve tails for both hot and cold water injections were consistently estimated at close to 1.5 for heat transport. By contrast, the tail slope for the salt tracer breakthrough curves is 3, even for simultaneous salt and heat injections. The temperature observations of the convergent tests performed in April 2019 and August 2019 were modeled using HydroGeoSphere. The model includes four observed fractures connecting the wells. As the hydrogeological situation between April and August 2019 changes dramatically, the model considers two saturated fractures in April and four saturated fractures in August 2019. A first parameterization of the model was determined, which allowed simulation of the observations and the slopes of the breakthrough curve tails. Results show that heating and cooling the porous medium from an injected highly permeable fracture is influenced by conduction (as expected), however, the conduction direction (i.e., from the fracture toward the matrix or vice‐versa) possibly influences the thermal recovery rate. Differences between a hot and cold water injection observed via analysis of breakthrough curves in the present study appear negligible at first glance, because they are small. Peak arrival times and the slopes of the breakthrough curve tails are the same for hot and cold injections. For thermal recovery, however, there are distinct differences, attributed to temperature influences on fluid properties (density and viscosity), and possibly to the temperature dependency of the thermal conductivity and specific heat capacity values. Although not clearly observed in the presented experiments, the impact of temperature gradient on fluid viscosity and density, as well as on heat parameters might be accentuated over larger spatial and temporal scales, such as in geothermal applications. Hence, a key finding of this study is the distinct characteristics of the hot vs. cold water injection experiments. The inverse condition for heat transport from fracture to matrix and matrix to fracture should be symmetrical, except due to the influences on variable density and viscosity and resultant flow dynamics and due to temperature‐induced changes of the thermal conductivity and specific heat capacity, possibly affecting the hot or cold plume. In addition, differences in fluid temperature may also temporarily induce changes in fracture aperture due to thermal expansion or contraction of the medium (e.g., Lima et al. 2019). This could indeed lead to differences in the behavior of the two plumes. However, whether the changes in fluid temperature induced here were enough to cause significant expansion or contraction of the medium was clearly beyond the scope of this study.

In summary, the results clearly show the usefulness of heat as a tracer and especially cold water injection in relatively warmer natural groundwaters. This confirms that temperature is an important and interesting, highly informative tracer for groundwater science. Cold water as a tracer has indeed a high potential to investigate aquifers showing a naturally high groundwater temperature. Attention should, however, be devoted to temperature effects on fluid properties which appear to alter flow and transport conditions and may be important when higher resolution interpretations are sought.

## Authors' Note

The authors do not have any conflicts of interest or financial disclosures to report.

## Data Availability

The Choutuppal EHP has benefited from BRGM and NGRI funding and INSU (Institut national des sciences de l'Univers) support within the H+ Observatory and OZCAR Research Infrastructure. The data of the present work are accessible online (http://hplus.ore.fr/en/hoffmann‐et‐al‐2021‐groundwater‐data). The field video from Selles et al. (2019) is accessible online (https://www.youtube.com/watch?v=cx6s4cGj1sc).
